# Untargeted and Targeted Blood Lipidomic Signature Profile of Gestational Alcohol Exposure

**DOI:** 10.3390/nu15061411

**Published:** 2023-03-15

**Authors:** Vishal D. Naik, Jayanth Ramadoss

**Affiliations:** 1 Department of Obstetrics & Gynecology, C.S. Mott Center for Human Growth and Development, School of Medicine, Wayne State University, Detroit, MI 48201, USA; 2 Department of Physiology, School of Medicine, Wayne State University, Detroit, MI 48201, USA

**Keywords:** FASD, pregnancy, alcohol, untargeted, targeted lipidomics

## Abstract

Alcohol consumption has a close relationship with blood lipid levels in a nonpregnant state, with a myriad of effects on the liver; however, little is known about the interaction of alcohol and lipids in the context of fetal alcohol spectrum disorders (FASD). We herein aimed to determine the effect of alcohol on the lipid profile in a pregnant rat model, with a focus on FASD. Dry blood spots (50 µL) were obtained from rat maternal blood collected on gestational day (GD) 20, two hours after the last binge alcohol exposure (4.5 g/kg, GD 5–10; 6 g/kg, GD 11–20). The samples were then analyzed using high-throughput untargeted and targeted lipid profiling via liquid chromatography-tandem mass spectrometry (LC-MS/MS). In untargeted lipidomics, 73 of 315 identified lipids were altered in the alcohol group compared to the pair-fed controls; 67 were downregulated and 6 were upregulated. In targeted analysis, 57 of the 260 studied lipid subspecies were altered, including Phosphatidylcholine (PC), Phosphatidylethanolamine (PE), Phosphatidylglycerol (PG), Phosphatidic Acid (PA), Phosphatidylinositol (PI), and Phosphatidylserine (PS); 36 of these were downregulated and 21 lipid subspecies were upregulated. These findings suggest alcohol-induced dysregulation of lipids in the maternal blood of rats and provide novel insights into possible FASD mechanisms.

## 1. Introduction

Alcohol’s teratogenic effects have been well documented and investigated extensively since the early 1970s. Alcohol consumption during pregnancy can lead to fetal alcohol spectrum disorders (FASD). This umbrella term includes a range of conditions in those who are exposed to prenatal alcohol [1,2]. Alcohol consumption has a close relationship with blood lipid levels in a non-pregnant state, with a myriad of effects on the liver; however, little is known about the interaction of alcohol and lipids in the context of FASD [3,4]. Understanding the dynamics of lipids and their homeostasis has recently attracted attention in the context of FASD as lipids utilize a variety of active and passive mechanisms to cross the placenta and reach the developing fetus [5,6].

Early pregnancy comprises a net anabolic lipid accumulation phase followed by a net catabolic phase in late pregnancy to meet the increasing metabolic demands of the growing fetus [7,8]. Alcohol has been reported to alter total lipid levels in pregnant animals and human tissues [9,10,11]. For instance, alcohol consumption during pregnancy significantly reduced the normal increases in the total serum lipids observed during pregnancy, including levels of total and low-density lipoproteins (LDL) cholesterol, LDL phospholipids, proteins, and total LDL [12]. Further, there is increasing evidence showing a relationship between the lipid rafts of cell membranes and alcohol. Lipid rafts are organized lipid microdomains containing nanoscale assemblies of sphingomyelins, cholesterol, and protein receptors that compartmentalize cellular processes affecting membrane signaling and protein trafficking [13]. Alcohol has been demonstrated to affect caveolar lipid rafts in pregnant ovine endothelial cells, and L1-mediated protein trafficking through lipid rafts in both in vivo and in vitro animal models [14,15]. 

The documentation of alcohol-induced lipid profile adaptations in pregnancy has been limited. We aimed to determine the effect of alcohol on the blood lipid profile in a pregnant rat model, using a two-step approach. First, a broad untargeted approach was performed, utilizing high-throughput liquid chromatography mass spectrometry (LC-MS) lipidomics, which has the potential to provide thousands of unique MS features with a unique mass-to-charge ratio (*m/z*) at a unique retention time, allowing for the identification of a wide range of lipids affected by maternal alcohol exposure. The second step involved a targeted approach, where selected phospholipid groups including Phosphatidic Acid (PA), Cardiolipin (CL), Phosphatidylethanolamine (PE), Phosphatidylcholine (PC), Phosphatidylglycerol (PG), Phosphatidylinositol (PI), and Phosphatidylserine (PS) were analyzed in the maternal blood to obtain a detailed understanding of the lipids and lipid-subspecies affected by alcohol consumption. We therefore hypothesized that binge alcohol exposure significantly alters the blood lipid abundance signature profile during pregnancy. Further, we also hypothesized that alcohol exposure during pregnancy will differentially alter the phospholipid subspecies levels in the maternal blood.

## 2. Materials and Methods

### 2.1. Treatment Groups and Alcohol Dosing Paradigm

All experimental procedures were in accordance with the National Institutes of Health guidelines (NIH Publication No. 85–23, revised 1996), with approval by the Animal Care and Use Committee at Texas A&M University. Timed pregnant Sprague–Dawley rats purchased from Charles River (Wilmington, MA, USA) arrived on GD 4 and were housed in a temperature-controlled room (23 °C) with a 12:12 h light-dark cycle. All dams were allowed to acclimatize for one day before handling, weighing, and assignment into experimental groups. A nutritional pair-fed control group and a binge alcohol group were utilized for lipidomic analysis. Binge alcohol group dams were acclimatized via a once-daily orogastric gavage of 4.5 g/kg ethanol (22.5% wt/v; peak BAC, 216 mg/dl) from GD 5 to 10, and advanced to a 6 g/kg alcohol dose from GD 11 to 20 (28.5% wt/v; peak BAC, 289 mg/dl). Daily feed intake of the alcohol rats was measured, and an equal amount of feed was given to pair-fed dams as a nutrition control. In addition, to control for the calories derived from alcohol, pair-fed control rats received a once-daily isocaloric maltose-dextrin. The exposure regimen utilized in this study was modeled after reported alcohol consumption patterns in pregnant women and FASD animal models [16,17,18]. 

### 2.2. Blood Collection

On GD 20, two hours after alcohol or maltose-dextrin administration, blood was collected from each dam via the tail (PF control n = 5, alcohol n = 5) in EDTA-coated tubes. A total of 5 aliquots of 50 μL blood from the tube was added to 903 ^TM^ Protein saver card (Whatman 903 Protein Saver Card; GE Healthcare Ltd., Pittsburgh, PA, USA) and allowed to dry before storing at 4 °C. A flow chart of the procedures is depicted in Figure S1.

### 2.3. Untargeted and Targeted Lipid Analysis

50 µL spots were then cut out, following the sample card guides, and placed in the bottom of 15 mL glass screw-cap tubes. A total of 175 μL water and 2 mL methanol was added to the samples. A methyl-tert-butyl ether (MTBE)-based liquid-liquid extraction was used. A pre-mixed solution of 625 µL methanol and 50 µL of a custom internal standard mixture was added to each sample, followed by 8.75 mL MTBE. After vigorous vortexing, the mixture was incubated on a tabletop shaker at 550 rpm at room temperature for one hour. Phase separation was induced by the addition of 2.2 mL water. The samples were shaken for 10 min and then centrifuged at 2000× *g* for 15 min. Following centrifugation, the upper organic phase of each sample was carefully removed using a Pasteur pipette and transferred into a fresh glass tube. The remaining aqueous phase was re-extracted with 2 mL of clean MTBE/methanol/water 10:3:2.5 (*v*:*v*:*v*). After vortexing and centrifuging as above, the organic phase was collected and combined with the initial organic phase. The extracted lipids were dried in a SpeedVac vacuum concentrator. The dried lipid extracts were reconstituted in 500 μL n-butanol/methanol 1:1 (*v/v*) and transferred into autosampler vials for analysis by LC-MS/MS. After analysis, all remaining extracts were dried again in a SpeedVac concentrator, resuspended in the initial conditions of solvents used for targeted phospholipid analysis, and analyzed using the corresponding method. 

### 2.4. Quality Control

Human plasma quality control samples (Cayman chemical; n = 5) were run throughout the sample sequence to assess instrument performance. Quality control samples were prepared from 10 µL human plasma by the same protocol, stored as dried lipid extracts under nitrogen at −80 °C, and reconstituted in n-butanol/methanol 1:1 (*v/v*) immediately before use. Aliquots were reconstituted for single use only. Instrument performance was verified to be within an acceptable range throughout the sample queue. Major lipid classes and their percent coefficients of variation are listed in Table 1. We commonly observed <15% CV (coefficients of variation) for all lipid classes.

### 2.5. Data Processing

Unprocessed LC-MS data contain several thousand unique MS features, defined as a unique mass-to-charge ratio (*m/z*) at a unique retention time. Feature detection, noise and artifact reduction, alignment, normalization, and lipid identification were analyzed using Lipostar software (Molecular Discovery). The specific data treatment steps applied to this sample set include the following: performed peak picking, smoothing, and retention time alignment to generate a list of all detected peaks with a unique *m/z* and retention time (features). A retention time window of from 1 to 32 min was applied to remove early and late eluting interferences. We removed background peaks that appeared in the blank solvent, removed peaks that did not contain MS/MS spectra (with the exception of fatty acids), removed peaks that did not present the correct isotope pattern, and removed noise and high-signal background (CODA algorithm). We performed automated lipid identification by querying the Lipid Maps database: this database included major lipid classes (phospholipids, sphingolipids, DAGs, TAGs, and sterols) for both positive- and negative-ion modes and was modified to include additional lipids (e.g., additional cardiolipins and some internal standards). The identification algorithm used the detected accurate *m/z* with a 5 ppm mass tolerance filter and the corresponding MS/MS spectrum to identify lipids and ranks the identifications via a 1-to-4-star scale of increasing confidence. Per standard practices, one-star-ranked identifications were excluded from the list of identified lipids. The integrated areas of all identified lipids were normalized to the integrated area of their corresponding class-specific internal standard within each sample. Identifications of the five most abundant molecular species in each class (sorted by their peak area ratio) were manually reviewed using MS/MS spectra and peak integration.

### 2.6. Statistical Analysis

Data from the targeted analysis of phospholipids were analyzed using MultiQuant 3.0 (Sciex, Redwood City, CA, USA). Both targeted and untargeted data are reported as the ratios of the integrated area for each analyte and the integrated area of the corresponding internal standard. Statistical difference was analyzed using Student’s *t*-test, and significance was established at *p* < 0.05. 

## 3. Results

To aid in the visualization of untargeted data, lipids were identified and classified in positive- and negative-ion modes utilizing normalized and identified peaks after applying filters to remove low-confidence identifications in Lipostar. Fatty acids were manually approved as they do not produce MS/MS spectra (Figures S2 and S4). 

### 3.1. Untargeted Lipidomic Analysis

**Figure 1:**
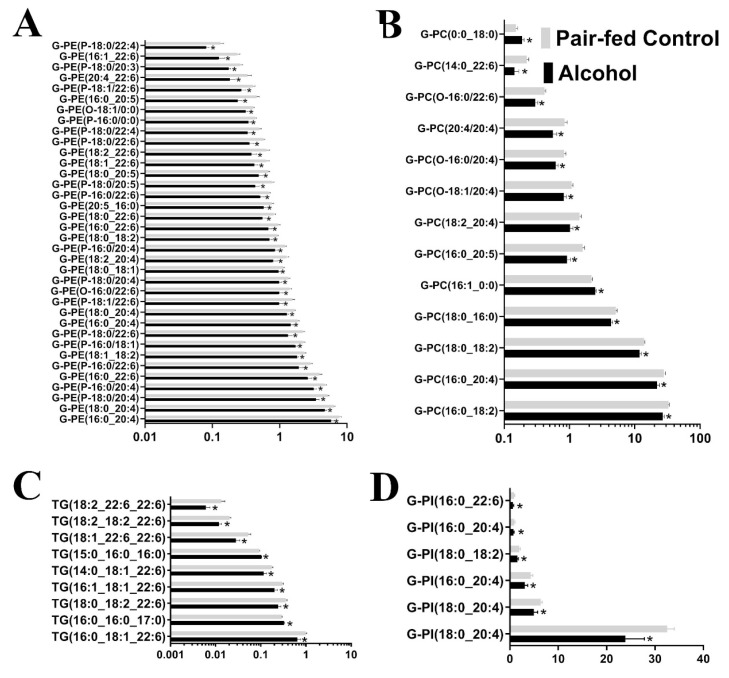
Significantly altered (*p* < 0.05) glycerophospholipids in the maternal blood following gestational alcohol exposure. A total of 73 lipids were affected by alcohol treatment. The four most affected lipid species were Glycerophosphoethanolamines (G-PE; **A**), Glycerophosphocholines (G-PC; **B**), Triacylglycerols (TG; **C**), and Glycerophosphoinositols (G-PI; **D**). *, *p* < 0.05.

A total of 1726 lipids with unique *m/z* charges and retention times were detected (Figures S3 and S5). An total of 315 lipids were identified with high confidence scores (>2) as determined by the identification software Lipostar (Table S1). In total, 73 lipids were affected by alcohol treatment. Of the 73 lipids affected, 67 were downregulated, and 6 were upregulated (Table 2 and Table S2). Lipid subclasses affected by binge alcohol exposure are shown in Figure 1. A further analysis of lipid pathway performed using LipidMaps showed Glycerophosphoserines (G-PS) to Glycerophosphoethanolamines (G-PE) as the most suppressed pathway (z-score: −0.696), while the conversion of G-PE to Glycerophosphocholines (G-PC) was the most active (z-score: 1.61; Figure S6).

### 3.2. Targeted Lipidomic Analysis

**Figure 2:**
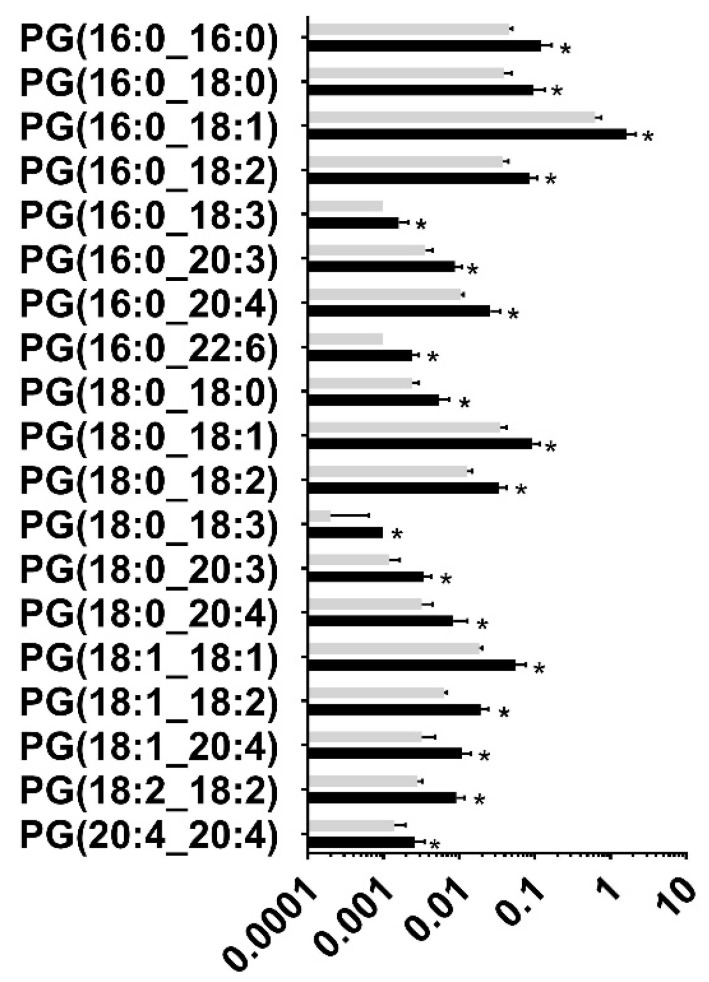
Significantly altered Phosphatidylglycerol subspecies in the maternal blood following gestational binge alcohol exposure. Targeted analysis of Phosphatidylglycerol (PG) was performed to identify PG levels in rat maternal blood following alcohol consumption during pregnancy. Nineteen subspecies were significantly altered (*p* < 0.05) in maternal blood of the alcohol-administered rat. Interestingly, all 19 subspecies were upregulated in the maternal blood of alcohol-administered rats. *, *p* < 0.05.

**Figure 3:**
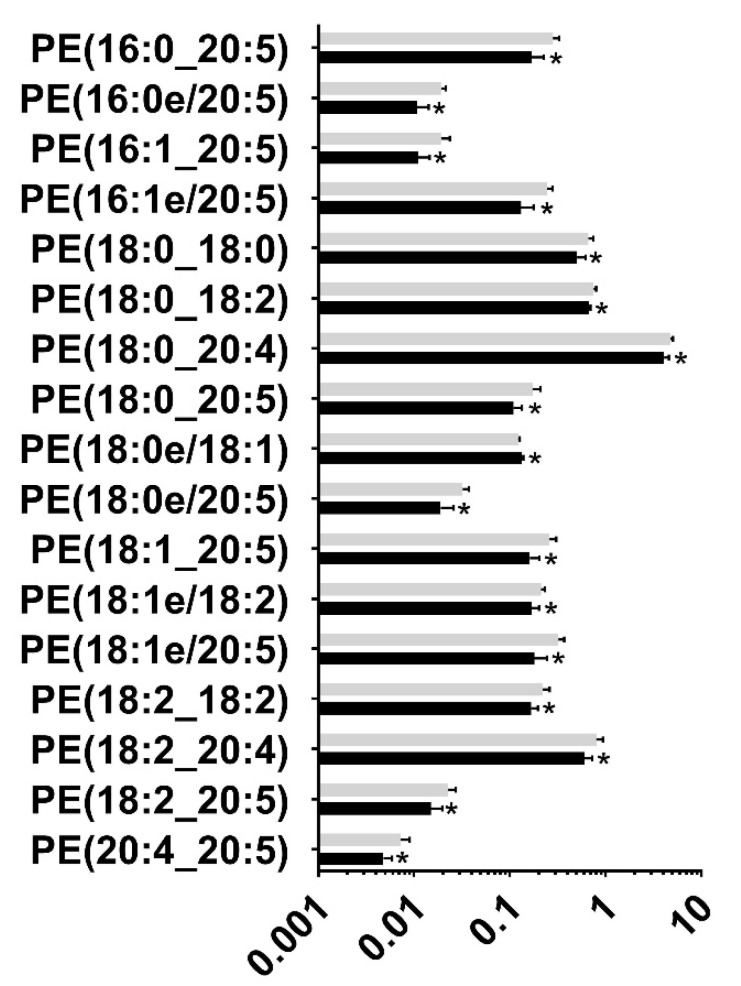
Significantly altered Phosphatidylethanolamine subspecies in the maternal blood following gestational alcohol exposure. Targeted analysis of Phosphatidylethanolamine (PE) was performed to identify PE levels in rat maternal blood following alcohol consumption during pregnancy. Seventeen subspecies were significantly altered (*p* < 0.05) in maternal blood of the alcohol-administered rat, of which 16 were downregulated and 1 was upregulated (PE 18:0e/18:1). *, *p* < 0.05.

**Figure 4:**
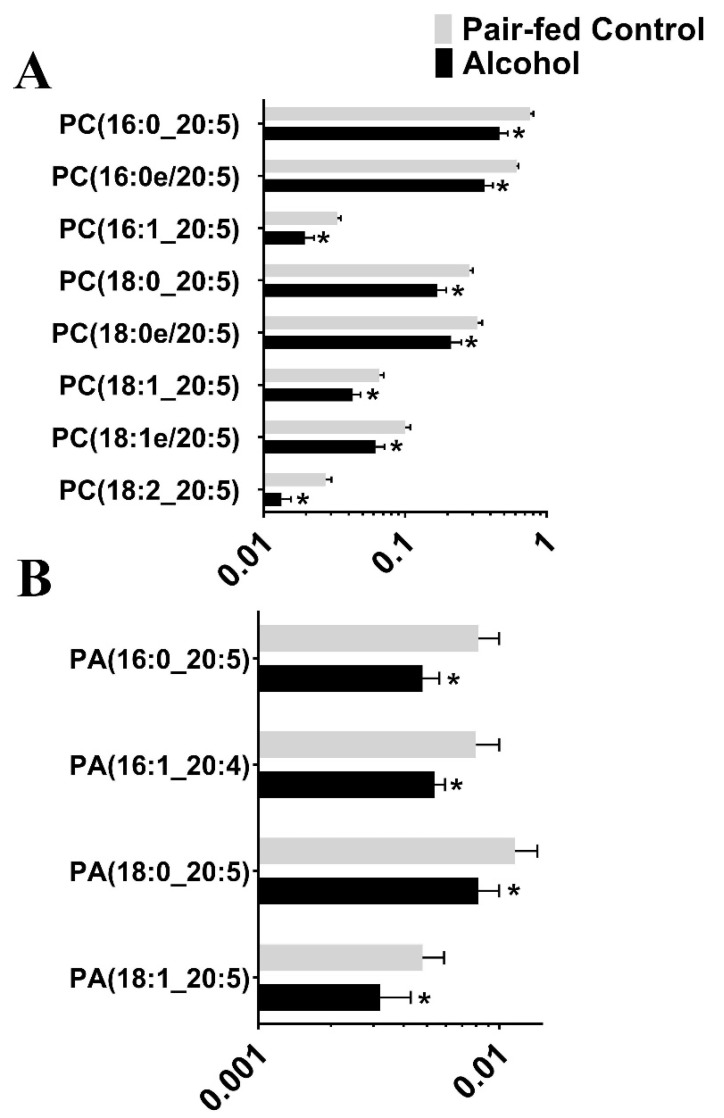
Significantly altered (*p* < 0.05) Phosphatidylcholine and Phosphatidic Acid subspecies in the maternal blood following gestational alcohol exposure. (**A**) Targeted analysis of Phosphatidylcholine (PC) and (**B**) Phosphatidic Acid (PA) was performed to identify PC and PA levels in rat maternal blood following alcohol consumption during pregnancy. *, *p* < 0.05.

**Figure 5:**
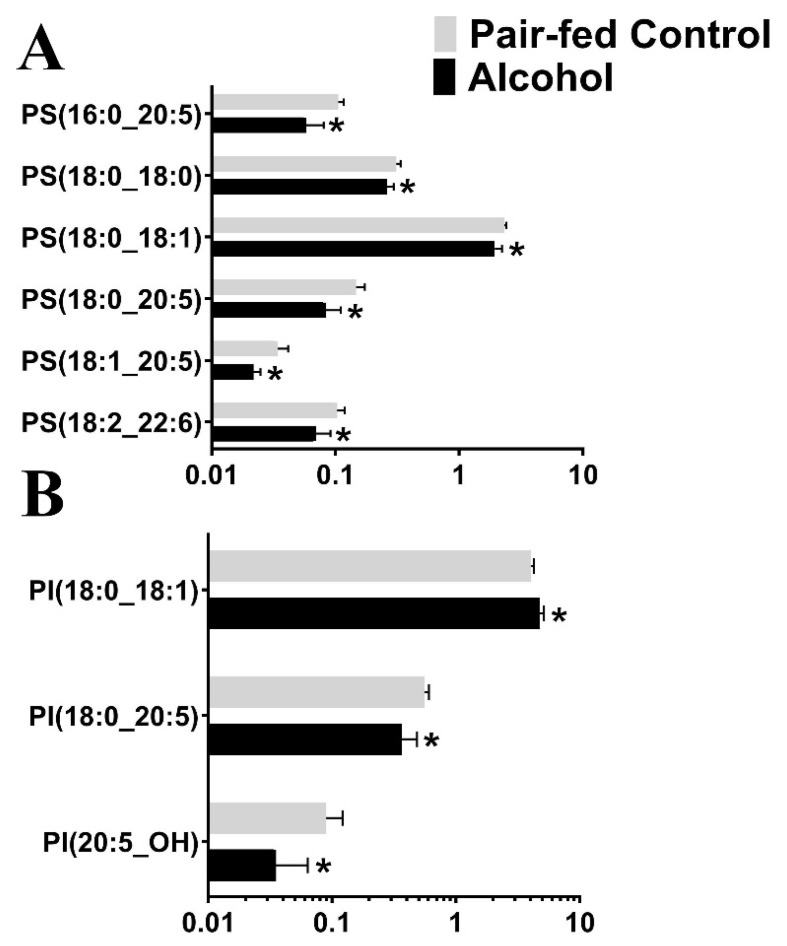
Significantly altered (*p* < 0.05) Phosphatidylserine and Phosphatidylinositol subspecies in the maternal blood following gestational alcohol exposure. (**A**) Targeted analysis of phosphatidylserine (PS) and (**B**) Phosphatidylinositol (PI) was performed to identify of PS and PI levels in rat maternal blood following alcohol consumption during pregnancy. *, *p* < 0.05.

Following untargeted lipid analysis, a targeted lipid analysis was performed to quantify specific lipid and lipid subspecies. Seven species of lipids were targeted for quantifying alcohol-induced changes to the lipid profile: Phosphatidic Acid (PA), Cardiolipin (CL), Phosphatidylethanolamine (PE), Phosphatidylcholine (PC), Phosphatidylglycerol (PG), Phosphatidylinositol (PI), and Phosphatidylserine (PS). A total of 342 lipid subspecies were analyzed, of which 260 lipids were detected. Surprisingly, cardiolipin, which is found in the mitochondrial membrane, was not detected in the targeted analysis, although a few of them were identified in the untargeted lipidomic analysis, probably due to the two different types of chromatography employed. Of the 260 lipid subspecies detected, 57 lipid subspecies were altered (Table 3 and Table S3). Of those 57 lipid subspecies altered, 36 were downregulated and 21 lipid subspecies were upregulated. Most notably, all Phosphatidylglycerol subspecies that were affected were upregulated (Figure 2). The second most altered species of lipid was Phosphatiylethanolamin (PE), with 16 of the 17 affected subspecies of PE being downregulated following chronic binge ethanol exposure (Figure 3). All subspecies of Phosphatidylcholine (PC) and Phosphatidic Acid (PA) that were altered due to gestational binge alcohol exposure were downregulated (Figure 4A,B). Six subspecies of Phosphatidylserine (PS) were altered with alcohol exposure, and all were downregulated (Figure 5A). Two of three subspecies of Phosphatidylinositol (PI) altered by alcohol were downregulated, and one was upregulated (Figure 5B). BioPAN analysis of the most suppressed pathways included the PE to PC (z-score: 1.394), PC to PS (z-score: 1.044), and PS to PE (z-score: 0.031) pathways. 

## 4. Discussion

This study utilized a two-pronged approach to identify gestational chronic binge alcohol effects on blood lipid levels in an FASD rat model. An untargeted approach was utilized to identify alcohol-induced changes in maternal lipid profile, followed by a targeted approach to identify specific lipids and lipid subspecies changes due to alcohol. Overall, there was a decrease in maternal blood lipid levels in alcohol-administered rats compared to those in the pair-fed control rats. Targeted lipid analysis revealed lipid-specific effects of alcohol. In normal pregnancy, hyperlipidemia is natural and expected during early pregnancy for the healthy development of the baby [19,20,21]. Low lipid levels have been associated with low birth weight and preterm birth [22]. The current study provides evidence of alcohol-induced dysregulation of lipids in the maternal blood of rats, which may be important in discerning alcohol’s effects on fetal development. 

In this study, we first aimed to identify individual molecular species and detect alcohol-induced alterations in the lipid profile in the maternal blood of pregnant rats. In a mammalian cell membrane, the most abundant phospholipid is G-PC, which constitutes about 50–60% of total phospholipids, followed by G-PE, which constitutes about 15–25% of total lipids [23,24]. G-PI, sterols, G-PS, and SL constitute about 5–10% of total phospholipids, each depending on the organelle location [23]. For example, G-PE is mostly located in the inner lipid bilayer of the plasma membrane, and abundantly found in the inner mitochondrial membrane [25]. G-PS is predominantly located in the endoplasmic reticulum, while sterols enrich the plasma membrane. In mammalian cells, these phospholipids are required for important cellular functions such as membrane protein folding, cell signaling, energy transport, and autophagy [26,27,28,29]. 

Normal human pregnancy is associated with hyperlipidemia, which is an adaptation to meet the increased energy needs of the mother and the developing fetus. Most lipid species, including G-PE, G-PC, and TG, were previously reported to significantly increase in the maternal plasma starting from the first trimester up to delivery [30]. The chronic binge alcohol exposure paradigm utilized in this study resulted in significant decreases in G-PE, G-PC, TG, and G-PI in the maternal blood. Interestingly, these lipids were also reported to be downregulated in the placenta and plasma of patients with pre-eclampsia [31,32]. The concentration of TG in the third trimester is a stronger predictor of birth weight than glucose-related parameters [33,34]. In this study, there was a significant decrease in seven of the nine subclasses of TG affected by alcohol. As it is well established that low birthweight is a common phenotype in FASD, our results are in agreement with previous studies directly correlating decreased TG levels with decreased fetal birth weight. 

Targeted analysis of lipids revealed the downregulation of various subspecies of PA, PC, PE, PI, and PS. PA is involved in several important functions, including cell signaling regulation and lipid biosynthesis. Studies utilizing pregnant rat models have demonstrated the accumulation of Phosphatidylethanol (PEth) in tissues and blood following alcohol exposure. PEth is formed only in the presence of ethanol by replacing PA through a transphosphatidylation reaction involving the enzyme Phospholipase D (PLD) and substrate Phosphatidylcholine (PC). In the liver, PC is synthesized via sequential methylation of PE, through the catalytic activity of Phosphatidylethanolamine N-methyltransferase (PEMT). In pregnant ewes, PC is reported to increase during early pregnancy and is essential for brain development. In the same study, PE, PI, and PS increased during late pregnancy [35]. In the uterine endometrium of guinea pigs, PC, PE, and TG are the main sources of arachidonic acid during prostaglandin synthesis, and there is a significant increase in arachidonic acid during late pregnancy. Further, there was a strong correlation reported between PE, PC, and DG and birth weights [36]; this suggests that decreases in PA, PC, PE, PI, and PS following alcohol exposure may negatively impact fetal development. 

An interesting observation in our study was the significant increase in 19 subspecies of PG in maternal blood following alcohol exposure. PG was the only lipid that was upregulated among all the subspecies that were significantly altered by alcohol. PG is the second most abundant lipid in lung surfactant and its levels in amniotic fluids are used to determine fetal lung maturity [37]. The major role of PG in mammalian membranes is to act as a precursor to cardiolipin, where two PG molecules combine to form a cardiolipin molecule and the reaction is catalyzed by the enzyme cardiolipin synthase [38]. Although we were unable to detect cardiolipin in this study, others have reported a decrease in mitochondrial cardiolipin levels following alcohol exposure in rats [39]. It is possible that alcohol interferes with this process, thus inhibiting cardiolipin synthesis, explaining the observed increase in PG levels in the rat maternal blood. Further, amniotic and plasma PG levels detected early in pregnancy have been linked to severe pregnancy complications including preeclampsia and gestational diabetes [40]. 

In conclusion, this study utilized a two-pronged approach using high-throughput untargeted and targeted lipidomic to identify lipid adaptations in rat maternal blood following alcohol exposure. There was a significant decrease in overall lipid levels in the alcohol-exposed maternal rat blood. Alcohol downregulated specific lipids that are shown to be critical for fetal development. This study provides novel insights into possible FASD mechanisms and provides evidence to further explore lipid pathways in FASD diagnosis, mechanisms, and treatment. 

## Figures and Tables

**Table 1:** Quality Control Precision showing percent coefficients of variation among various lipids measured using human plasma quality control samples to validate instrument performance.

Lipid Class	%CV
Diacylglycerols	13%
Sphingomyelins	3%
Sterols	7%
Triacylglycerols	8%
Glycerophosphocholines	5%
Glycerophosphoethanolamines	8%
Glycerophosphoinositols	11%
Glycerophosphoserines	10%

**Table 2:** Class of lipids and their corresponding lipid subclass number, which was significantly altered following alcohol exposure in an untargeted lipidomic analysis.

Lipid Class	Number of Lipid Subclasses Affected by Alcohol	Effect
Glycerophosphoethanolamines (G-PE)	37	Downregulated
Glycerophosphocholines (G-PC)	13	11 Downregulated, 2 upregulated
Triacylglycerols (TG)	9	7 Downregulated, 2 upregulated
Phosphosphingolipids (SL)	6	5 Downregulated, 1 upregulated
Glycerophosphoinositols (G-PI)	6	Downregulated
Glycerophosphoserines (G-PS)	1	Downregulated
Sterols	1	Upregulated
Total	73	

**Table 3:** Species of lipids and their corresponding lipid subspecies number, which was significantly altered by alcohol following targeted lipidomic analysis.

Lipid Species	Lipid Subspecies Altered by Alcohol	Effect
Phosphatidic Acid (PA)	4	Downregulated
Phosphatidylcholine (PC)	8	Downregulated
Phosphatidylethanolamine (PE)	17	16 Downregulated, 1 upregulated
Phosphatidylglycerol (PG)	19	Upregulated
Phosphatidylinositol (PI)	3	2 Downregulated, 1 upregulated
Phosphatidylserine (PS)	6	Downregulated

## Data Availability

Not applicable.

## Supplementary Materials

The following supporting information can be downloaded at: https://www.mdpi.com/article/10.3390/nu15061411/s1.
