# Supplementary material for: Untargeted and Targeted Blood Lipidomic Signature Profile of Gestational Alcohol Exposure
Source: Nutrients. 2023 Mar 15;15(6):1411. doi: 10.3390/nu15061411 (PMC10051993; doi:10.3390/nu15061411)
Supplement: Supplementary file 1 [file nutrients-15-01411-s001.zip › nutrients-2272506-supplementary.pdf]

Supplementary Materials

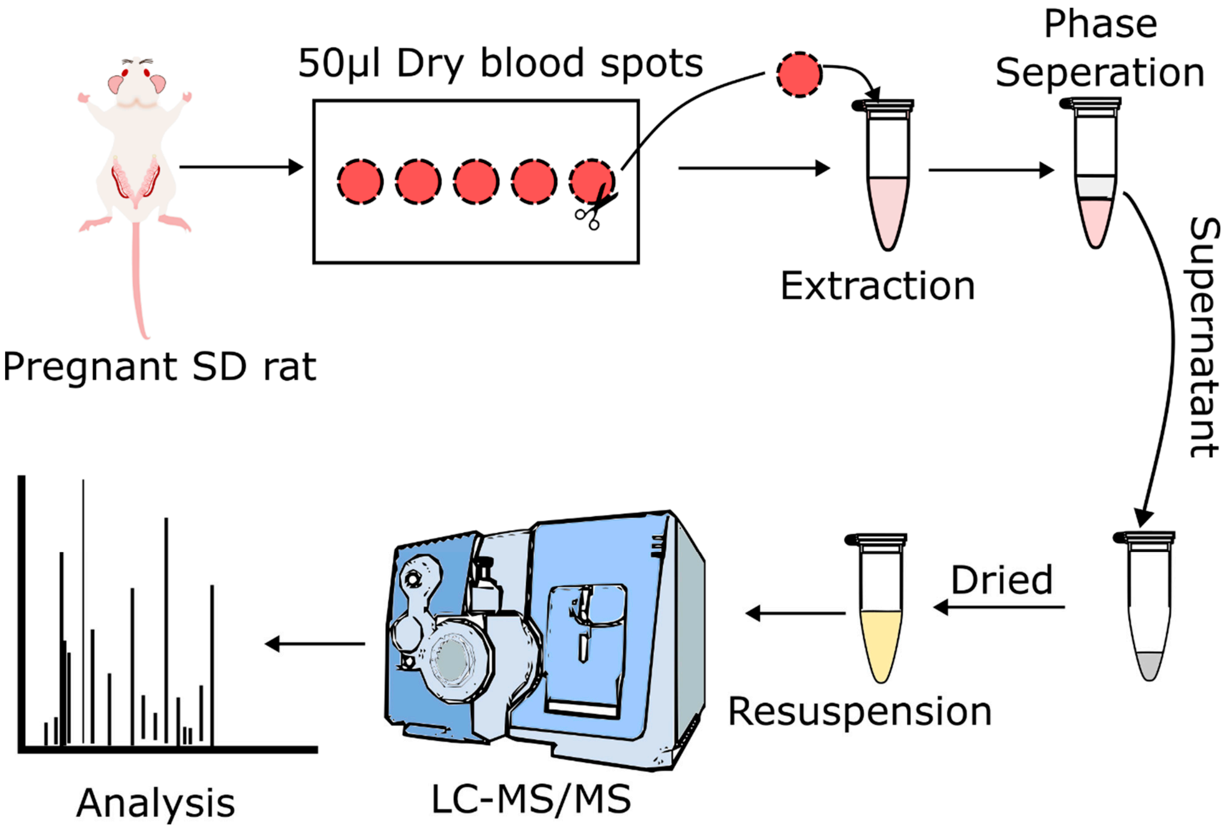

Figure S1. Schematic overview the lipidomic procedure.

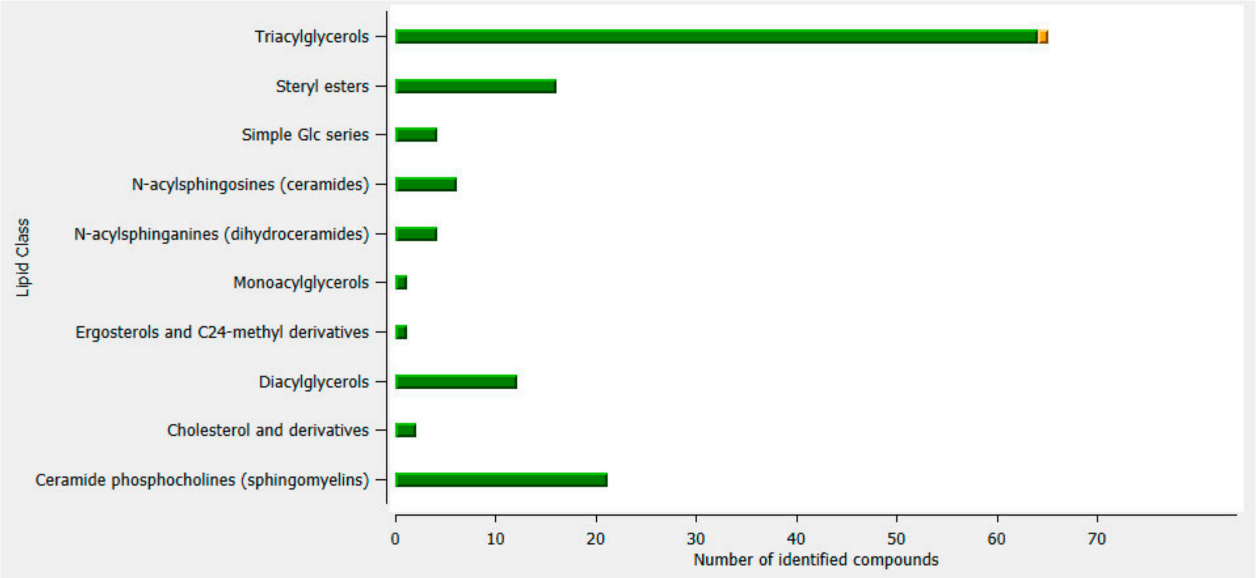

Figure S2. *Summary of molecular species identified in positive-ion mode.* This plot provides a histogram of the number of identified positive-ion features.

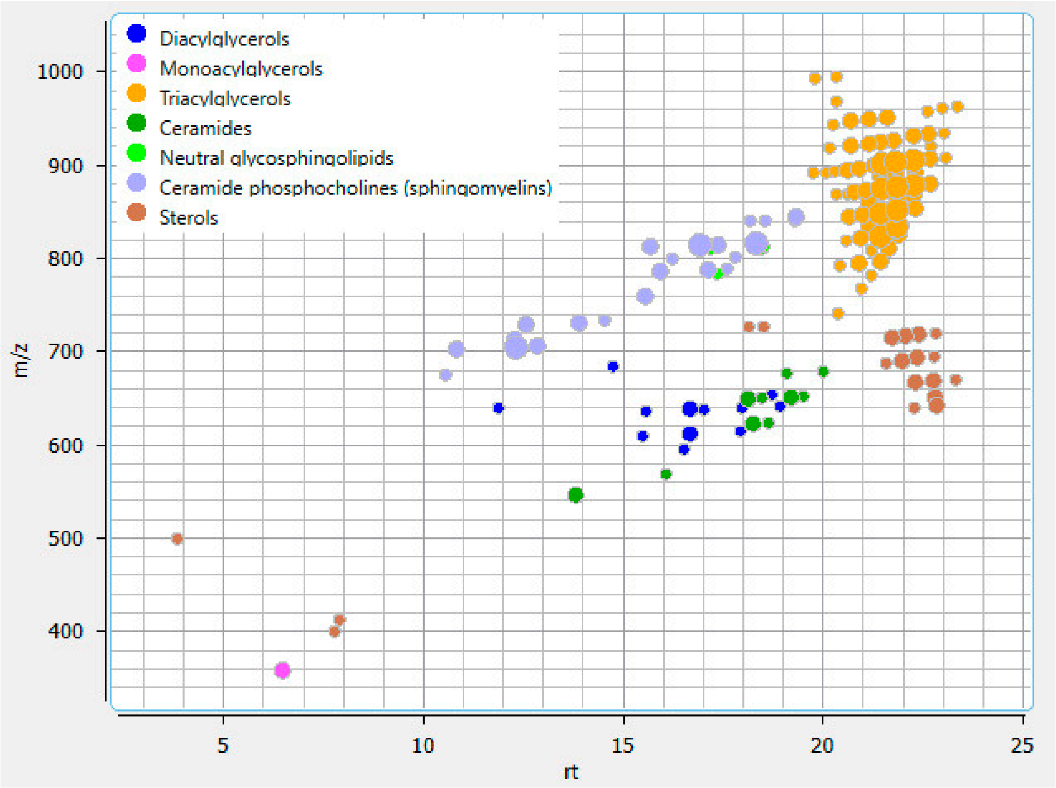

Figure S3. *Mass-to-charge ratio (m/z) vs retention time plot of positive ions. A* visual overview of the identified peaks in positive-ion mode from all samples combined, color coded by lipid class. The size of the spots is proportional to their relative abundance as peak area ratio (analyte/internal standard).

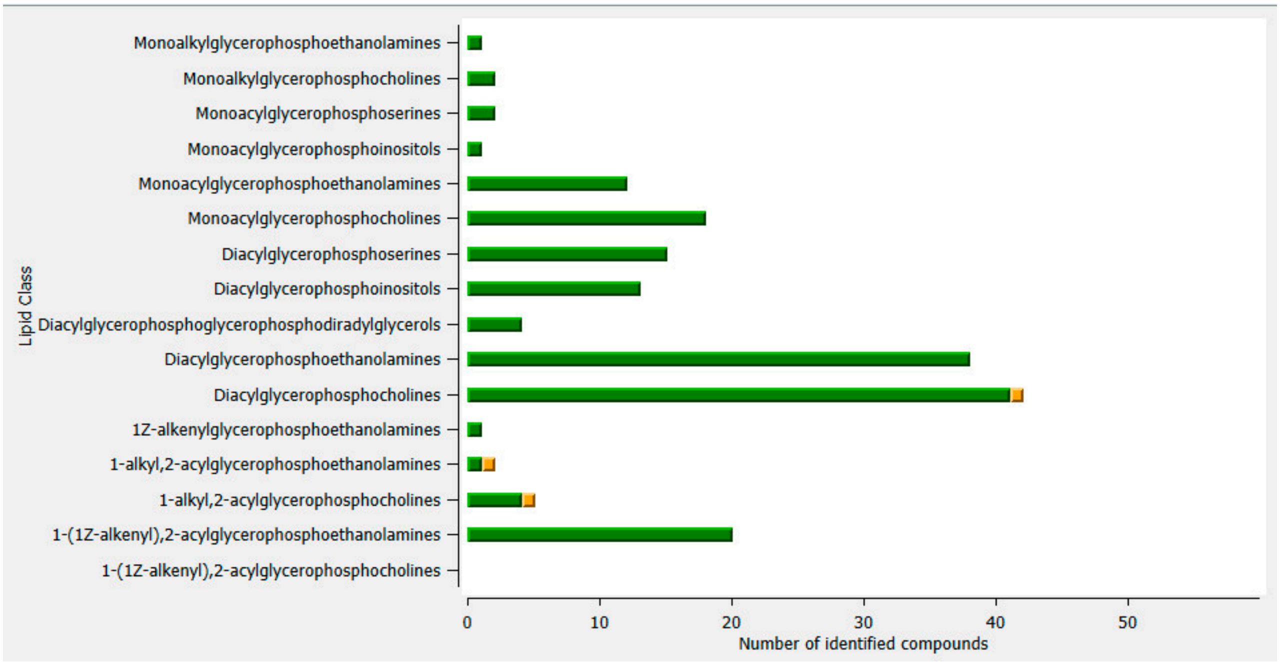

Figure S4. *Summary of molecular species identified in negative-ion mode.* This plot provides a histogram of the number of identified negative-ion features.

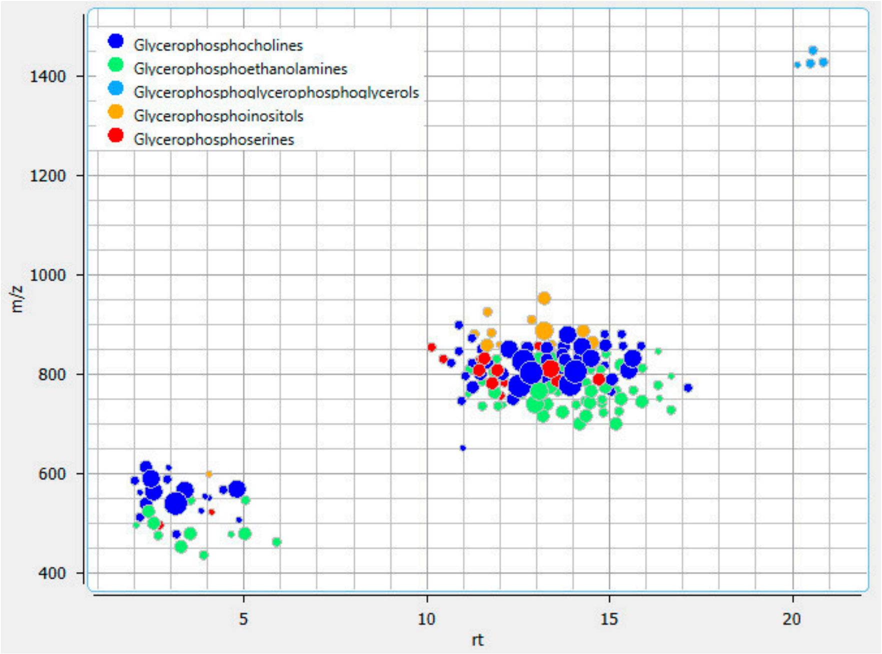

Figure S5. *Mass-to-charge ratio (m/z) vs retention time plot of negative ions.* A visual overview of the identified peaks in negative-ion mode from all samples combined, color coded by lipid class. The size of the spots is proportional to their relative abundance as peak area ratio (analyte/internal standard).

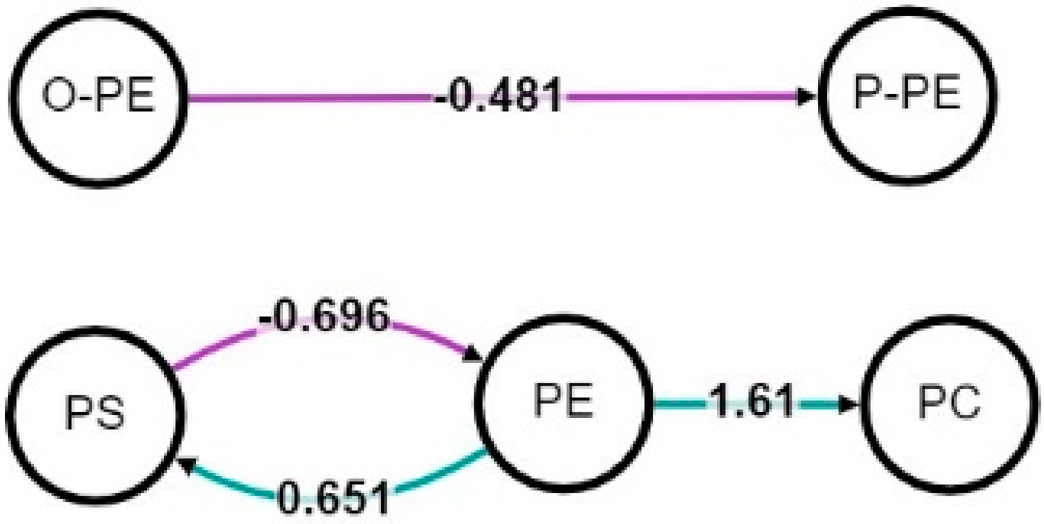

Figure S6. *Most active- and suppressed reaction and pathways.* A visual overview of most active and suppressed reaction and pathways between Pair-fed control and alcohol group as analyzed using LipidMaps.

Table S1. Numbers of Identified Molecular Species by Class and Subclass (Lipid Maps Classification).

**Numbers of Identified Molecular Species by Class and Subclass (Lipid Maps Classification).**	
**Ceramides**	**9**
N-acylsphinganines (dihydroceramides)	4
N-acylsphingosines (ceramides)	5
**Diacylglycerols (DG)**	**11**
**Fatty Acids and Conjugates**	**12**
Fatty acids	9
Hydroxy fatty acids	3
**Glycerophosphocholines (PC)**	**65**
1-alkyl,2-acylglycerophosphocholines	5
Diacylglycerophosphocholines	41
Monoacylglycerophosphocholines	17
Monoalkylglycerophosphocholines	2
**Glycerophosphoethanolamines (PE)**	**76**
1-(1Z-alkenyl),2-acylglycerophosphoethanolamines	22
1-alkyl,2-acylglycerophosphoethanolamines	2
1Z-alkenylglycerophosphoethanolamines	1
Diacylglycerophosphoethanolamines	38
Monoacylglycerophosphoethanolamines	12
Monoalkylglycerophosphoethanolamines	1
**Glycerophosphoglycerophosphoglycerols (CL)**	**5**
Diacylglycerophosphoglycerophosphodiradylglycerols	5
**Glycerophosphoinositols (PI)**	**15**

Diacylglycerophosphoinositols	14
Monoacylglycerophosphoinositols	1
**Glycerophosphoserines (PS)**	**17**
Diacylglycerophosphoserines	15
Monoacylglycerophosphoserines	2
**Monoradylglycerols**	**1**
**Neutral glycosphingolipids**	**4**
Simple Glc series	4
**Ceramide phosphocholines (sphingomyelins) (SM)**	**20**
**Sterols**	**16**
Cholesterol and derivatives	2
Ergosterols and C24-methyl derivatives	1
Steryl esters	13
**Triacylglycerols (TG)**	**64**
**Total**	**315**

Table S2. Fold change and P-values in untargeted lipidomics.

**Molecular Species**	**Lipid Class**	**Lipid Subclass**	**p-Value**	**Fold Change**	**Obervation**
(20R22R)-2022-dihydroxycholesterol	Sterols	Cholesterol and derivatives	0.012964	0.72743348	Upregulated
LysoPE(0:0_20:5)	Glycerophosphoethanolamines	Monoacylglycerophosphoethanolamines	0.0398	-0.34734184	Downregulated
PC(0:0_18:0)	Glycerophosphocholines	Monoacylglycerophosphocholines	0.045674	0.24535438	Upregulated
PC(14:0_22:6)	Glycerophosphocholines	Diacylglycerophosphocholines	0.02639	-0.35053119	Downregulated
PC(16:0_18:2)	Glycerophosphocholines	Diacylglycerophosphocholines	0.013561	-0.18777102	Downregulated
PC(16:0_20:4)	Glycerophosphocholines	Diacylglycerophosphocholines	0.048485	-0.20923542	Downregulated
PC(16:0_20:5)	Glycerophosphocholines	Diacylglycerophosphocholines	0.003056	-0.42270341	Downregulated
PC(16:1_0:0)	Glycerophosphocholines	Monoacylglycerophosphocholines	0.037247	0.14392127	Upregulated
PC(18:0_16:0)	Glycerophosphocholines	Diacylglycerophosphocholines	0.045463	-0.15145998	Downregulated
PC(18:0_18:2)	Glycerophosphocholines	Diacylglycerophosphocholines	0.037057	-0.14580984	Downregulated
PC(18:2_20:4)	Glycerophosphocholines	Diacylglycerophosphocholines	0.027674	-0.28097996	Downregulated
PC(20:4/20:4)	Glycerophosphocholines	Diacylglycerophosphocholines	0.04594	-0.33552687	Downregulated
PC(O-16:0/20:4)	Glycerophosphocholines	1-alkyl,2-acylglycerophosphocholines	0.017634	-0.25560261	Downregulated
PC(O-16:0/22:6)	Glycerophosphocholines	1-alkyl,2-acylglycerophosphocholines	0.009509	-0.27661442	Downregulated
PC(O-18:1/20:4)	Glycerophosphocholines	1-alkyl,2-acylglycerophosphocholines	0.023766	-0.246618	Downregulated

PE(16:0_20:4)	Glycerophosphoethanolamines	Diacylglycerophosphoethanolamines	0.044354	-0.24356478	Downregulated
PE(16:0_20:4)	Glycerophosphoethanolamines	Diacylglycerophosphoethanolamines	0.049754	-0.20929542	Downregulated
PE(16:0_20:5)	Glycerophosphoethanolamines	Diacylglycerophosphoethanolamines	0.004828	-0.47743286	Downregulated
PE(16:0_22:6)	Glycerophosphoethanolamines	Diacylglycerophosphoethanolamines	0.030672	-0.32819892	Downregulated
PE(16:0_22:6)	Glycerophosphoethanolamines	Diacylglycerophosphoethanolamines	0.040031	-0.28477406	Downregulated
PE(16:1_22:6)	Glycerophosphoethanolamines	Diacylglycerophosphoethanolamines	0.013283	-0.46351708	Downregulated
PE(18:0_18:1)	Glycerophosphoethanolamines	Diacylglycerophosphoethanolamines	0.041335	-0.1524376	Downregulated
PE(18:0_18:2)	Glycerophosphoethanolamines	Diacylglycerophosphoethanolamines	0.011499	-0.23996974	Downregulated
PE(18:0_20:4)	Glycerophosphoethanolamines	Diacylglycerophosphoethanolamines	0.004107	-0.25884887	Downregulated
PE(18:0_20:4)	Glycerophosphoethanolamines	Diacylglycerophosphoethanolamines	0.004724	-0.22722437	Downregulated
PE(18:0_20:5)	Glycerophosphoethanolamines	Diacylglycerophosphoethanolamines	0.036235	-0.2780752	Downregulated
PE(18:0_22:6)	Glycerophosphoethanolamines	Diacylglycerophosphoethanolamines	0.010885	-0.32176478	Downregulated
PE(18:1_18:2)	Glycerophosphoethanolamines	Diacylglycerophosphoethanolamines	0.020208	-0.21192099	Downregulated
PE(18:1_22:6)	Glycerophosphoethanolamines	Diacylglycerophosphoethanolamines	0.047854	-0.33152371	Downregulated
PE(18:2_20:4)	Glycerophosphoethanolamines	Diacylglycerophosphoethanolamines	0.024248	-0.36132739	Downregulated
PE(18:2_22:6)	Glycerophosphoethanolamines	Diacylglycerophosphoethanolamines	0.041618	-0.40113414	Downregulated
PE(20:4_22:6)	Glycerophosphoethanolamines	Diacylglycerophosphoethanolamines	0.035615	-0.44986043	Downregulated

PE(20:5_16:0)	Glycerophosphoethanolamines	Diacylglycerophosphoethanolamines	0.030955	-0.24061563	Downregulated
PE(O-16:0/22:6)	Glycerophosphoethanolamines	1-alkyl,2-acylglycerophosphoethanolamines	0.030865	-0.29160853	Downregulated
PE(O-18:1/0:0)	Glycerophosphoethanolamines	Monoalkylglycerophosphoethanolamines	0.007393	-0.23108903	Downregulated
PE(P-16:0/0:0)	Glycerophosphoethanolamines	1Z-alkenylglycerophosphoethanolamines	0.029322	-0.20061354	Downregulated
PE(P-16:0/18:1)	Glycerophosphoethanolamines	1-(1Z-alkenyl),2-acylglycerophosphoethanolamines	0.032119	-0.22589886	Downregulated
PE(P-16:0/20:4)	Glycerophosphoethanolamines	1-(1Z-alkenyl),2-acylglycerophosphoethanolamines	0.012984	-0.31033166	Downregulated
PE(P-16:0/20:4)	Glycerophosphoethanolamines	1-(1Z-alkenyl),2-acylglycerophosphoethanolamines	0.010928	-0.28311475	Downregulated
PE(P-16:0/22:6)	Glycerophosphoethanolamines	1-(1Z-alkenyl),2-acylglycerophosphoethanolamines	0.027079	-0.32547637	Downregulated
PE(P-16:0/22:6)	Glycerophosphoethanolamines	1-(1Z-alkenyl),2-acylglycerophosphoethanolamines	0.047911	-0.25926571	Downregulated
PE(P-18:0/20:3)	Glycerophosphoethanolamines	1-(1Z-alkenyl),2-acylglycerophosphoethanolamines	0.012356	-0.32018721	Downregulated
PE(P-18:0/20:4)	Glycerophosphoethanolamines	1-(1Z-alkenyl),2-acylglycerophosphoethanolamines	0.005856	-0.33531187	Downregulated
PE(P-18:0/20:4)	Glycerophosphoethanolamines	1-(1Z-alkenyl),2-acylglycerophosphoethanolamines	0.011697	-0.27707214	Downregulated
PE(P-18:0/20:5)	Glycerophosphoethanolamines	1-(1Z-alkenyl),2-acylglycerophosphoethanolamines	0.00315	-0.43788169	Downregulated
PE(P-18:0/22:4)	Glycerophosphoethanolamines	1-(1Z-alkenyl),2-acylglycerophosphoethanolamines	0.007697	-0.32657583	Downregulated
PE(P-18:0/22:4)	Glycerophosphoethanolamines	1-(1Z-alkenyl),2-acylglycerophosphoethanolamines	0.025418	-0.37745622	Downregulated

PE(P-18:0/22:6)	Glycerophosphoethanolamines	1-(1Z-alkenyl),2-acylglycerophosphoethanolamines	0.006499	-0.39577869	Downregulated
PE(P-18:0/22:6)	Glycerophosphoethanolamines	1-(1Z-alkenyl),2-acylglycerophosphoethanolamines	0.005225	-0.37582648	Downregulated
PE(P-18:1/22:6)	Glycerophosphoethanolamines	1-(1Z-alkenyl),2-acylglycerophosphoethanolamines	0.018287	-0.35844567	Downregulated
PE(P-18:1/22:6)	Glycerophosphoethanolamines	1-(1Z-alkenyl),2-acylglycerophosphoethanolamines	0.041576	-0.32612134	Downregulated
PI(16:0_20:4)	Glycerophosphoinositols	Diacylglycerophosphoinositols	0.005886	-0.28668304	Downregulated
PI(16:0_20:4)	Glycerophosphoinositols	Diacylglycerophosphoinositols	0.043766	-0.2255854	Downregulated
PI(16:0_22:6)	Glycerophosphoinositols	Diacylglycerophosphoinositols	0.020681	-0.30522366	Downregulated
PI(18:0_18:2)	Glycerophosphoinositols	Diacylglycerophosphoinositols	0.024881	-0.20172803	Downregulated
PI(18:0_20:4)	Glycerophosphoinositols	Diacylglycerophosphoinositols	0.001674	-0.26621007	Downregulated
PI(18:0_20:4)	Glycerophosphoinositols	Diacylglycerophosphoinositols	0.006659	-0.22088965	Downregulated
PS(18:0_18:1)	Glycerophosphoserines	Diacylglycerophosphoserines	0.033526	-0.12873491	Downregulated
SM(d16:1/24:1)	Phosphosphingolipids	Ceramide phosphocholines (sphingomyelins)	0.000163	-0.26465766	Downregulated
SM(d17:1/24:1)	Phosphosphingolipids	Ceramide phosphocholines (sphingomyelins)	0.041579	0.16872072	Upregulated
SM(d18:0/22:0)	Phosphosphingolipids	Ceramide phosphocholines (sphingomyelins)	0.008956	-0.20435751	Downregulated
SM(d18:1/16:1)	Phosphosphingolipids	Ceramide phosphocholines (sphingomyelins)	0.001477	-0.14477802	Downregulated
SM(d24:1/18:1)	Phosphosphingolipids	Ceramide phosphocholines (sphingomyelins)	0.008518	-0.19497704	Downregulated

SM(d26:1/18:1)	Phosphosphingolipids	Ceramide phosphocholines (sphingomyelins)	0.008063	-0.26221638	Downregulated
TG(14:0_18:1_22:6)	Triradylglycerols	Triacylglycerols	0.028989	-0.3373549	Downregulated
TG(15:0_16:0_16:0)	Triradylglycerols	Triacylglycerols	0.008912	0.1138037	Upregulated
TG(16:0_16:0_17:0)	Triradylglycerols	Triacylglycerols	0.007499	0.12622925	Upregulated
TG(16:0_18:1_22:6)	Triradylglycerols	Triacylglycerols	0.029267	-0.34209496	Downregulated
TG(16:1_18:1_22:6)	Triradylglycerols	Triacylglycerols	0.028843	-0.32733661	Downregulated
TG(18:0_18:2_22:6)	Triradylglycerols	Triacylglycerols	0.033527	-0.31243992	Downregulated
TG(18:1_22:6_22:6)	Triradylglycerols	Triacylglycerols	0.036958	-0.46729967	Downregulated
TG(18:2_18:2_22:6)	Triradylglycerols	Triacylglycerols	0.006824	-0.41903541	Downregulated
TG(18:2_22:6_22:6)	Triradylglycerols	Triacylglycerols	0.038133	-0.53840984	Downregulated

Table S3. Fold change and P-values in targeted lipidomics.

Component Name	P-value	Fold Change	Observation
PA(16:0_20:5)	0.004879315	-0.414634146	Downregulated
PA(16:1_20:4)	0.023067617	-0.325	Downregulated
PA(18:1_20:5)	0.049735563	-0.333333333	Downregulated
PE(16:0_20:5)	0.007511166	-0.404661017	Downregulated
PE(16:1_20:5)	0.012034365	-0.427083333	Downregulated
PE(16:0e/20:5)	0.001828191	-0.4375	Downregulated
PE(16:1e/20:5)	0.001940685	-0.469255663	Downregulated
PE(18:0_18:0)	0.03805855	-0.239215686	Downregulated
PE(18:0_18:2)	0.007746768	-0.116662238	Downregulated
PE(18:0_20:4)	0.041206605	-0.13469405	Downregulated
PE(18:0_20:5)	0.012861419	-0.362790698	Downregulated
PE(18:0e/18:1)	0.028582587	0.081833061	Upregulated
PE(18:0e/20:5)	0.009465852	-0.409937888	Downregulated
PE(18:1_20:5)	0.007487479	-0.376822717	Downregulated
PE(18:1e/18:2)	0.039205499	-0.203021719	Downregulated
PE(18:1e/20:5)	0.003679739	-0.440940012	Downregulated
PE(18:2_18:2)	0.036143541	-0.246846847	Downregulated
PE(18:2_20:4)	0.048753052	-0.244867301	Downregulated
PE(18:2_20:5)	0.040247574	-0.330357143	Downregulated
PE(20:4_20:5)	0.033730391	-0.333333333	Downregulated

PC(16:0_20:5)	0.005599484	-0.393558523	Downregulated
PC(16:1_20:5)	0.006270164	-0.409638554	Downregulated
PC(16:0e/20:5)	0.00250644	-0.400327869	Downregulated
PC(18:0_20:5)	0.005949119	-0.403100775	Downregulated
PC(18:0e/20:5)	0.035129931	-0.352544451	Downregulated
PC(18:1_20:5)	0.013834941	-0.351515152	Downregulated
PC(18:1e/20:5)	0.019542988	-0.382	Downregulated
PC(18:2_20:5)	0.004675328	-0.510948905	Downregulated
PG(16:0_16:0)	0.007120571	1.630434783	Upregulated
PG(16:0_18:0)	0.017171256	1.476683938	Upregulated
PG(16:0_18:1)	0.003240316	1.62221515	Upregulated
PG(16:0_18:2)	0.003528504	1.251336898	Upregulated
PG(16:0_18:3)	0.039968524	0.6	Upregulated
PG(16:0_20:3)	0.001109672	1.444444444	Upregulated
PG(16:0_20:4)	0.004680048	1.452830189	Upregulated
PG(16:0_22:6)	0.000446351	1.4	Upregulated
PG(18:0_18:0)	0.014063722	1.25	Upregulated
PG(18:0_18:1)	0.000614993	1.664772727	Upregulated
PG(18:0_18:2)	0.001030603	1.666666667	Upregulated
PG(18:0_18:3)	0.003949773	4	Upregulated
PG(18:0_20:3)	0.001164968	1.833333333	Upregulated

PG(18:0_20:4)	0.038170949	1.625	Upregulated
PG(18:1_18:1)	0.004730356	2.02173913	Upregulated
PG(18:1_18:2)	0.000502474	2	Upregulated
PG(18:1_20:4)	0.002060314	2.375	Upregulated
PG(18:2_18:2)	0.000763792	2.285714286	Upregulated
PG(20:4_20:4)	0.033730391	0.857142857	Upregulated
PI(18:0_18:1)	0.009366305	0.166945566	Upregulated
PI(18:0_20:5)	0.011172497	-0.338854879	Downregulated
PI(20:5_OH)	0.03119884	-0.606584821	Downregulated
PS(16:0_20:5)	0.002668482	-0.446969697	Downregulated
PS(18:0_18:0)	0.033077894	-0.158944659	Downregulated
PS(18:0_18:1)	0.018227462	-0.16382282	Downregulated
PS(18:0_20:5)	0.005581885	-0.434013605	Downregulated
PS(18:1_20:5)	0.008125641	-0.362573099	Downregulated
PS(18:2_22:6)	0.023500536	-0.321705426	Downregulated
